# Characterization of a Type 1 Metallothionein Gene from the Stresses-Tolerant Plant *Ziziphus jujuba*

**DOI:** 10.3390/ijms160816750

**Published:** 2015-07-23

**Authors:** Mingxia Yang, Fan Zhang, Fan Wang, Zhigang Dong, Qiufen Cao, Mingchang Chen

**Affiliations:** 1 The Institute of Loess Plateau, Shanxi University, Taiyuan 030006, China; E-Mails: ymx20051@163.com (M.Y.); mcchensx@sohu.com (M.C.); 2 Pomology Institute of Shanxi Academy of Agricultural Sciences, Taigu 030815, China; E-Mail: gssdzg@163.com; 3 Institute of Botany, Jiangsu Province and Chinese Academy of Sciences, Nanjing 210014, China; E-Mail: zhangfan_cau@126.com; 4 Jinguo Museum of Shanxi Province, Linfen 043400, China; E-Mail: wangfan2015_1@126.com; 5 Biotechnology Research Center of Shanxi Academy of Agricultural Sciences, Taiyuan 030031, China; 6 Department of Agriculture Shanxi Province, Taiyuan 030002, China

**Keywords:** cadmium, metallothionein, salt tolerance, *Ziziphus jujube*, ZjMT

## Abstract

Plant metallothioneins (MTs) are a family of low molecular weight, cysteine-rich, and metal-binding proteins, which play an important role in the detoxification of heavy metal ions, osmotic stresses, and hormone treatment. Sequence analysis revealed that the open-reading frame (ORF) of *ZjMT* was 225 bp, which encodes a protein composed of 75 amino acid residues with a calculated molecular mass of 7.376 kDa and a predicated isoelectric point (pI) of 4.83. ZjMT belongs to the type I MT, which consists of two highly conserved cysteine-rich terminal domains linked by a cysteine free region. Our studies showed that *ZjMT* was primarily localized in the cytoplasm and the nucleus of cells and *ZjMT* expression was up-regulated by NaCl, CdCl_2_ and polyethylene glycol (PEG) treatments. Constitutive expression of *ZjMT* in wild type *Arabidopsis* plants enhanced their tolerance to NaCl stress during the germination stage. Compared with the wild type, transgenic plants accumulate more Cd^2+^ in root, but less in leaf, suggesting that *ZjMT* may have a function in Cd^2+^ retension in roots and, therefore, decrease the toxicity of Cd^2+^.

## 1. Introduction

Heavy metals are essential for plant growth and development [1], however, excessive levels of essential as well as non-essential metals, such as Cadmium (Cd), are toxic to plants, causing a wide range of deleterious effects [2]. Cd^2+^ is a type of non-essential element and is taken up by plant roots and causes growth retardation [3]. Low concentration of Cd^2+^ in the rhizosphere can cause alterations in many physiological processes, including carbohydrate metabolism [4], nitrogen metabolism [5], photosynthesis [6], and therefore damage the nucleolus and membrane ATPase activity of plant cells [7]. In order to maintain metal homeostasis, plants have evolved numerous ways to mitigate detrimental effects of excessive metals ions, such as metal-chelating proteins metallothionein (MT).

The MTs are a class of low-molecular (6–7 kDa) cysteine (Cys)-rich proteins that bind heavy metals [8,9], and were first reported as a cadmium binding protein in the cortex of horse kidney [10]. This protein not only has effects on detoxification of heavy metals like cadmium and mercury [11], regulation of the homeostasis of essential metals including zinc and copper [12,13], but also has functions like protecting reactive oxygen species [14,15] and DNA damage [16], in animals, plants and microorganisms. A large number of cysteine residues in MTs are able to bind a variety of metals by the formation of mercaptide bonds [17]. Based on the distribution of Cys residues in their N- and C-terminal regions, plant MTs have been classified into four types, MT1, MT2, MT3 and MT4 [18,19]. Each type of MT exhibits a distinct spatial and temporal expression pattern in plant tissues during development and possibly has different functions. Type 1 MT genes are predominantly expressed in both leaves and roots, whereas type 2 MT genes are expressed in primarily in leaves, stems, and developing seed [20,21,22,23]. Type 3 MT genes are expressed in leaves or in ripening fruits [24], and the expression of type 4 MT genes are reported not only in seed, but also detected in reproductive organs and vegetative tissues [25,26]. The genes encoding the MTs have been identified and cloned from many plant species, including *Arabidopsis* [21], wheat [27], soybean [28], rice [29] and tomato [30], and increasing evidence suggests that plant MTs are also play an important role in physiological processes, including fruit ripening [31], root development, embryo germination [32], suberization [33] and response to multiple abiotic stresses [34]. Previous studies showed that, type 1 MT was required for Cd^2+^ and Cu^2+^ tolerance and accumulation [35,36], maintaining Zn^2+^ homeostasis, confer the adaptability of plant to drought stress and scavenging reactive oxidant species (ROS) [14,37].

Chinese jujube is a unique and economically important fruit tree, and has a long cultivation history in China. Moreover, it is well known for its high tolerance to stresses, such as cold, drought and high salinity, although the mechanisms underlying such stresses are still unknown. In this study, *ZjMT*, encoding a type I metallothionein, was cloned from Chinese jujube (*Ziziphus jujuba* Mill) full-length cDNA libraries, and expression pattern of *ZjMT* was identified in response to NaCl, CdCl_2_ and PEG treatments. In order to examine the function of *ZjMT*, an expression vector carrying the *ZjMT* gene driven by the cauliflower mosaic virus 35S (CaMV 35S) promoter was introduced into *Arabidopsis thaliana* genomes by the *Agrobacterium-*mediated transformation method. Transgenic plants showed tolerance to NaCl and CdCl_2_ stresses, and the Cd^2+^ was accumulated in roots and showed decreased accumulation in leaves.

## 2. Results

### 2.1. ZjMT Encodes a Protein with a Metallothionein (MT) Domain

**Figure 1:**
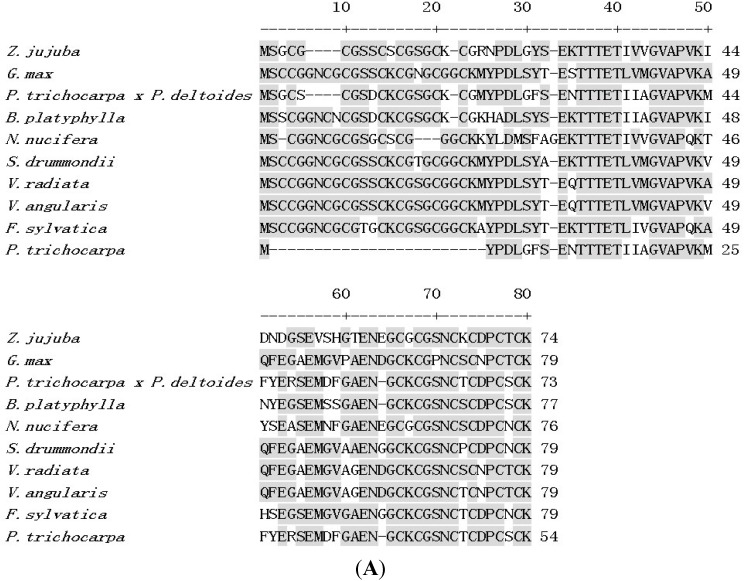

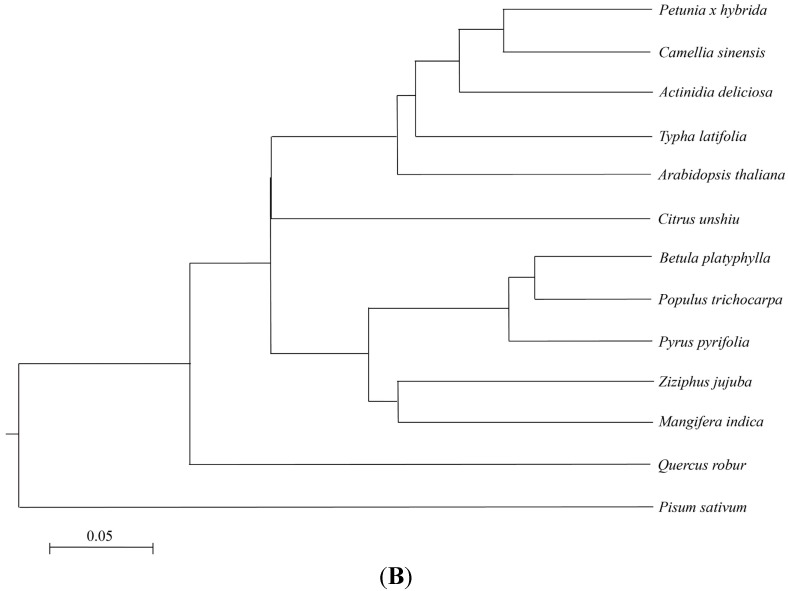
Multiple alignment of *ZjMT* and phylogenetic analysis. (**A**) Multiple alignments of MT proteins from selected species. Identical amino acid residues are highlighted in gray; (**B**) Phylogenetic analysis of MT domains from different species. All of the proteins used in the phylogenetic tree came from database of NCBI. The corresponding accession numbers of the names are as follows: *Petunia x hybrida* (AAG36945.1), *Camellia deliciosa* (ABD97257.1), *Actinidia deliciosa* (P43390.1), *Typha latifolia* (AAK28022.1), *Arabidopsis thaliana* (CAA44630.1), *Citrus unshiu* (BAA31561.1), *Betula platyphlla* (AAY166439.1), *Populus trichocarpa* (EEF07605.1), *Pyrus pyrifolia* (BAA96449.1), *Ziziphus jujuba* (AB513130), *Mangifera indica* (ACD69680.1), *Quercus robur* (CAE12162.1), and *Pisum sativum* (P20830.1).

*ZjMT* (GenBank No. AB513130) was obtained by screening jujube full-length cDNA libraries. The *ZjMT* cDNA is 225 bp in length and encodes a polypeptide of 74 amino acid residues and with a predicted molecular mass of 7.376 kDa. The deduced amino acid sequence analysis indicated that ZjMT contains highly conserved cysteine-rich domains in its N- and C-terminal respectively and a cysteine-free region between them, which was the common feature of the Type 1 MT proteins reported in other plants. With the BLASTN search from the NCBI database, the deduced amino acid sequence showed homology with counterpart Type I MT family members from other plant species (Figure 1A). Phylogenetic analysis revealed that ZjMT was clustered in the same clade with *Mangifera indica*, but distinct from *Pisum sativum* (Figure 1B). The proteins used in the alignment and phylogenetic tree all had an MT domain and were obtained by database searching in NCBI.

### 2.2. ZjMT Is a Potential Stress-Related Gene

**Figure 2:**
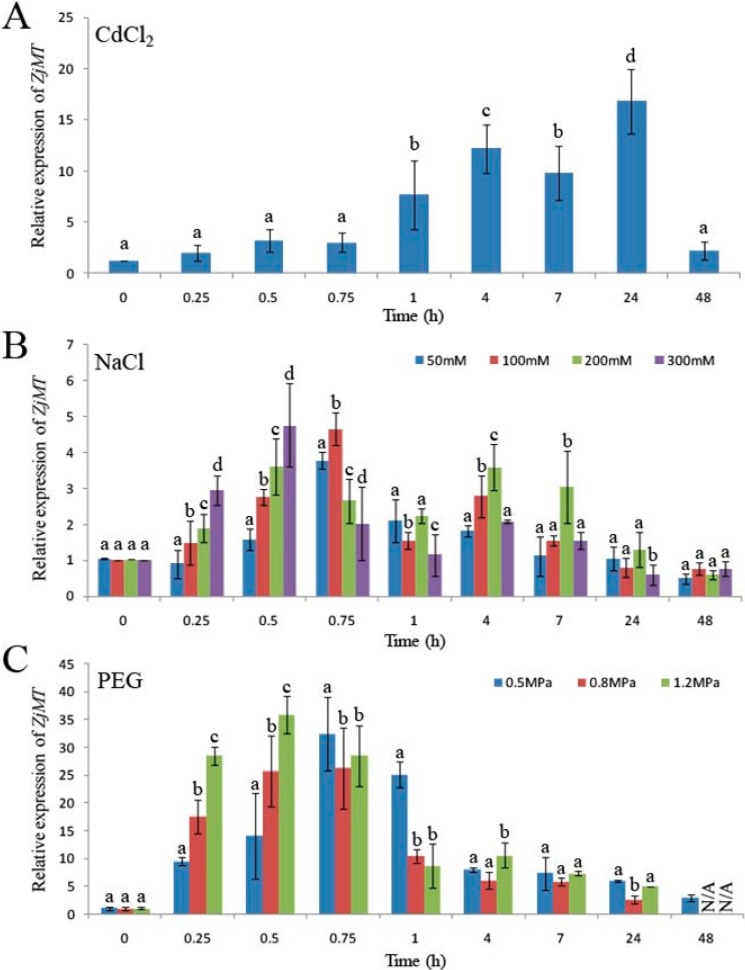
The expression patterns of *ZjMT*. The relative expression levels of *ZjMT* gene in leaves under CdCl_2_ (**A**), NaCl (**B**) and PEG (**C**) stress were measured using qRT-PCR. Six-week-old *Z. jujuba* young seedlings were treated with 100 mM CdCl_2_, 50, 100, 200 and 300 mM NaCl, and 10% PEG 6000 under different conditions at indicated time points. Different letters (a–d) indicate statistically significant differences between means at *p* < 0.05 (Student’s *t*-test). N/A: Not applicable. Standard errors were calculated from three biological replicates in which *ZjH3* (an *actin* gene, accession number EU916201) transcripts were used as internal controls. The 2^−ΔΔ*C*t^ method was used to measure the relative expression levels of the target gene in stressed and non-stressed leaves. Error bars represent standard error.

To identify whether *ZjMT* could be induced by heavy metal or other abiotic stresses, the expression profiles of *ZjMT* in *Z. jujuba* young seedlings under CdCl_2_, NaCl and PEG treatments were investigated using quantitative RT-PCR. *ZjMT* expression was significantly activated by CdCl_2_, NaCl and PEG stresses. The transcripts level of *ZjMT* increased at 0.25 h after CdCl_2_ treatment, reached a peak at 24 h, and then declined at 48 h (Figure 2A). *ZjMT* transcript level reached a peak at 0.75 h when the young seedlings were under 50 and 100 mM NaCl treatments, however at 0.5 h, it reached the peak under 200 and 300 mM NaCl treatments (Figure 2B). Similarly, the *ZjMT* transcript level reached a peak at 0.25 and 0.75 h under 1.2 MPa PEG treatments and 0.5 and 0.8 MPa PEG treatments, respectively (Figure 2C).

### 2.3. Subcelluar Localization of ZjMT

**Figure 3:**
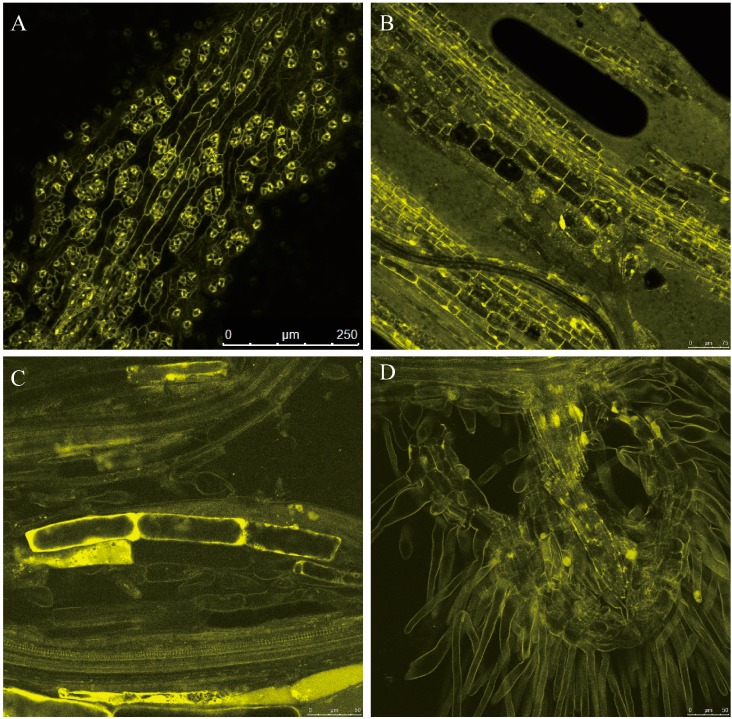
ZjMT is localized to cytoplasm and nucleus. ZjMT-YFP fusion proteins were constitutively expressed under control of the CaMV 35S promoter in *Arabidopsis* and observed with a laser scanning confocal microscope. Subcellular localization of ZjMT in *Arabidopsis* leaf epidermal cells (**A**); stem (**B**); roots (**C**) and root hairs (**D**). Scale bar = 250 μm (**A**), 75 μm (**B**), 50 μm (**C**,**D**).

To investigate the localization of ZjMT, the 35S:ZjMT-YFP plasmid was constructed and transformed into *Arabidopsis* by the floral dipping method. Homozygous transgenic lines were used for localization analysis. Firstly, we analyzed the localization of the ZjMT-YFP fusion protein in epidermal cells; it was primarily localized in the cytoplasm of the stomata guard cells (Figure 3A). In addition, the fluorescence could also be detected in the cytoplasm and nucleus in stem and roots, respectively (Figure 3B–D).

### 2.4. Constitutive Expression of ZjMT in Arabidopsis Enhances Their High Salinity Salt Tolerance

**Figure 4:**
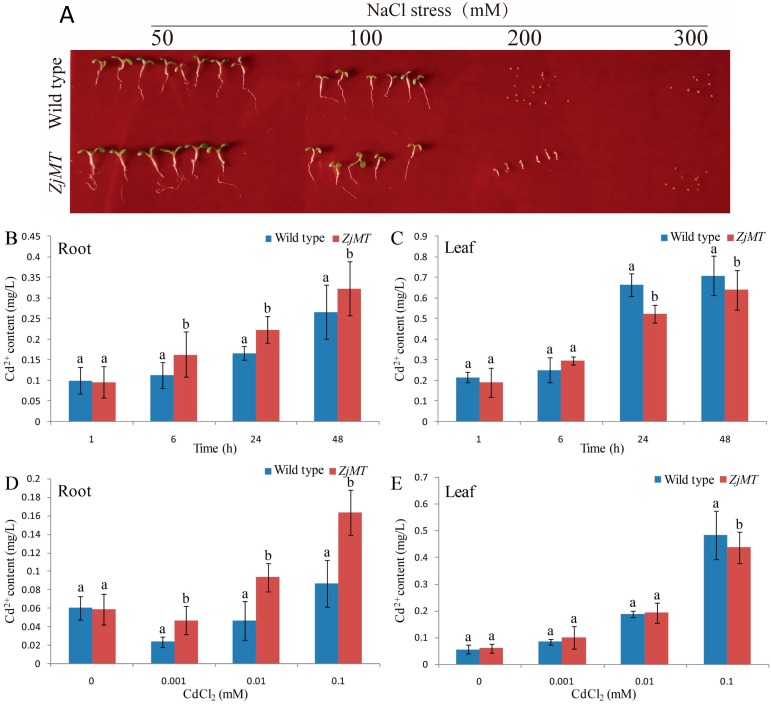
High salinity assays of *ZjMT* transgenic plants and the concentrations of Cd^2+^ in roots and leaves of wild type and transgenic *Arabidopsis* plants. (**A**) Phenotypic comparison of root lengths. Wild type and transgenic seeds were germinated and grown on MS medium with 50, 100, 200 or 300 mM NaCl for 7 days. The concentrations of Cd^2+^ in the roots (**B**) and leaves (**C**) of wild type and transgenic plants exposed to 0.1 mM CdCl_2_ with indicated time points. Cd^2+^ content in the roots (**D**) and leaves (**E**) of wild type and transgenic plants treated with various concentrations of CdCl_2_ for 24 h. Different letters in (**B**–**E**) indicate statistically significant differences between means at *p* < 0.05 (Student’s *t*-test).

We examined the role of *ZjMT* in NaCl stress responses, under normal conditions, no significant difference was observed between transgenic and wild type plants (data not shown). Although the cotyledon greening rate was similar between transgenic and wild type plants, the radicle emergence of transgenic seedlings was slightly higher than wild type in the presence of 50 mM NaCl medium. Furthermore, the radicle emergence of transgenic seedlings increased significantly compared to those of the wild type plants on the medium containing 100 mM NaCl, and the wild type seedlings were failed to develop radicle on the medium containing 200 mM NaCl. The wild type and transgenic seeds were both failed to germinate when the NaCl concentration reach to 300 mM (Figure 4). These results indicate that constitutive expression of *ZjMT* leads to enhanced tolerance in transgenic seedlings under salt stress and that *ZjMT* might act as a positive regulator to salt stress.

### 2.5. Cd^2+^ Accumulation and Distribution in Transgenic Plants

To explore whether constitutive expression of *ZjMT* influences endogenous Cd^2+^ content, the concentrations of Cd^2+^ in transgenic plants and wild type plants were measured. Although the concentration of Cd^2+^ in leaves of transgenic plants and wild type plants gradually increased after CdCl_2_ treatment, the transgenic plant accumulated less Cd^2+^ in leaves compared to wild type (Figure 4C). However, the root Cd^2+^ concentrations for transgenic plants were higher than those of WT plants (Figure 4B). In addition, similar results were observed after various concentrations of CdCl_2_ treatment (Figure 4D,E).

## 3. Discussion

Heavy-metal contamination is a great environmental concern globally, and the risk posed to humans is increasing. Metallothioneins (MTs) are Cys-rich proteins, which are involved in the metal tolerance of diverse living organisms. Although many studies have revealed the roles of MTs in plants in response to diverse metal stresses, the function of plant MTs remain poorly understood [38].

In this study, the *ZjMT* cDNA was cloned from *Ziziphus jujube* full-length cDNA libraries and determined as type-I MT based on the protein sequence alignment. Phylogenetic analysis also revealed that *ZjMT* shared high similarity of cysteine residue levels with other species (Figure 1). Previous research results of MT subcellular location showed that BjMT2 was localized in the cytoplasm of tobacco leaf cells, and AtMT4a and AtMT4b were both localized in cytoplasm, nucleus and membrane of *Arabidopsis* hypocotyls cells [39,40]. In our work, we found that the ZjMT was located in cytoplasm and nucleus (Figure 3). Microarray analysis indicated that MT transcripts were significantly up-regulated under salt and drought conditions in rice and barley [41,42]. In our study, the expression of *ZjMT* was generally induced after CdCl_2_ stress (Figure 2A), and the transcripts levels were also influenced by NaCl and PEG treatments (Figure 2B,C). To gain additional insight into the function of *ZjMT* in these stress responses, we evaluated the effect of salt stress on the growth of transgenic seedlings. On MS medium supplemented with NaCl, the wild type seedlings grew slowly, and failed to germination on the medium containing 200 mM NaCl, while the transgenic seedlings still develop radicles (Figure 4A). In addition, under CdCl_2_ stress, the transgenic plants exhibit increased accumulation of Cd^2+^ in roots and decreased the accumulation in leaves, whereas the accumulation of Cd^2+^ were increased both in roots and leaves in wild type plants (Figure 4B–E). In previous study, Cd^2+^ is taken up by plant roots and caused growth retardation [4], however further studies are needed to answer the underlying mechanisms of Cd^2+^ accumulation in different tissues. Furthermore, overexpression of plant MT genes increased Cd^2+^, Cu^2+^ and Zn^2+^ accumulation in transgenic plants [43], and type-I MT genes were more abundantly expressed in roots [20], according to these results we propose that *ZjMT* likely has a function in retention of Cd^2+^ in roots and decreased the Cd^2+^ toxic to leaves.

## 4. Experimental Section

### 4.1. Stress Treatments and Real-Time Polymerase Chain Reaction (PCR) Analyses

*Z. jujuba* Mill “Hupingzao” was used in this study and its seeds were germinated and grown in a greenhouse under controlled conditions: temperature at 25 ± 1 °C, a relative humidity of 65%–70%, and light density of ~2500 Lux at 12:12 h dark/light circle. For CdCl_2_ treatment, the six-week-old seedlings were transferred to ½ MS liquid medium (pH = 6.0) containing 100 mM CdCl_2_. For NaCl treatment, the seedlings were transferred to ½ MS liquid medium containing 50, 100, 200 or 300 mM NaCl. For PEG treatment, seedlings were transferred to ½ MS liquid medium containing 20% PEG (molecular weight 6000) under 0.5, 0.8, or 1.2 MPa [44]. The leaves of seedlings were harvested at indicated time points, and were snap-frozen in liquid nitrogen and stored at −80 °C before RNA isolation. *ZjH3*, an *actin* gene (accession number EU916201) transcripts were used as internal controls. The relative level of gene expression was detected using the 2^−ΔΔ*C*t^ method.

### 4.2. Sequence Analysis of ZjMT

The conserved domains of MT from *Z. jujube*, *G. max*, *P. trichocarpa x P. deltoids*, *B. platyphylla*, *N. nucifera*, *Sdrummondii*, *V. radiate*, *V. angularis*, *F. sylvatica* and *P. trichocarpa* were aligned using the ClustalX program (version 1.83) with default parameters. The phylogenetic tree was constructed using the neighbor-joining (NJ) method in MEGA (version 5.05) [45]. Bootstrap analysis was performed using 1000 replicates in MEGA to evaluate the reliability of different phylogenetic groups.

### 4.3. Subcellular Localization

The open reading frame (ORF) of *ZjMT* was cloned into *pEarleyGate-103* vector [46], which contained the yellow fluorescent protein (YFP) reporter gene, to generate a *ZjMT-YFP* fusion construct under the control of the CaMV 35S promoter. *Arabidopsis* plants were transformed by *Agrobacterium*-mediated floral dip. YFP fluorescence was observed under a confocal laser scanning system (Nikon, Tokyo, Japan), and examined at 514 nm (excitation) using an argon laser with an emission band of 515–530 nm.

### 4.4. Generation of Transgenic Plants

*ZjMT* cDNA was cloned into the vector under the control of CaMV 35S promoter. The construct was introduced into *Agrobacterium tumefaciens* GV3101 strain cells and then transferred into wild type *Arabidopsis* (ecotype *Columbia*) plants by floral infiltration [47]. The seeds of T_0_ generation were harvested and sown in soil, and 10-day-old seedlings of T_1_ plants were screened by spraying with 0.05% (*v*/*v*) phosphinothricin (ppt) solution. The survival transformants (T_1_) were confirmed by PCR amplification of *ZjMT*. The T_2_ seeds were planted on MS [48] agar medium containing 10 mg/L ppt and the transgenic lines with a 3:1 (resistant:sensitive) segregation ratio were selected to produce T_3_ seeds. The T_3_ lines displaying 100% ppt resistance were considered homozygous and used for further experiments.

### 4.5. Stress Treatments

For salt assays on plates, wild type and transgenic lines were planted on MS agar medium with various concentrations of NaCl for three days before being placed at a controlled environment. After 10 days, the phenotypes of the plants were examined and pictures were taken.

For CdCl_2_ experiments, two-week-old transgenic and wild type plants were planted in MS medium with various concentrations of CdCl_2_ for 24 h and then measured the Cd^2+^ content of roots and leaves.

### 4.6. Measurement of Cd^2+^ Content

For measurement of Cd^2+^ content in plant tissues, the seedlings of *Arabidopsis* wild type and transgenic plants were planted. After 14 days, seedlings were treated with 0.1 mM CdCl_2_ for 1, 6, 24, and 48 h. Roots and rosette leaves were excised carefully to determine their Cd^2+^ content. After 24 h at 105 °C, the dry weight was measured. The resulting dry matter was dissolved in nitric and perchloric acid (4:1) on a muffle furnace at 175 °C for 3 h. When the liquid became limpid, the Cd^2+^ content of the samples was determined with an atomic absorption spectrophotometer.

## 5. Conclusions

In conclusion, overexpression of *ZjMT* in *Arabidopsis* positively has a function in retention of Cd^2+^ in roots and decreased the Cd^2+^ toxic to leaves and enhances the salt tolerance of *Arabidopsis*. Therefore, *ZjMT* can be used as a candidate gene to improve stress tolerance by genetic transformation in crops.
